# Truxene Functionalized Star-Shaped Non-fullerene Acceptor With Selenium-Annulated Perylene Diimides for Efficient Organic Solar Cells

**DOI:** 10.3389/fchem.2021.681994

**Published:** 2021-05-12

**Authors:** Kaiwen Lin, Boming Xie, Zhenfeng Wang, Qingwu Yin, Yuehui Wang, Chunhui Duan, Fei Huang, Yong Cao

**Affiliations:** ^1^Department of Materials and Food, Zhongshan Institute, University of Electronic Science and Technology of China, Zhongshan, China; ^2^State Key Laboratory of Luminescent Materials and Devices, Institute of Polymer Optoelectronic Materials and Devices, South China University of Technology, Guangzhou, China

**Keywords:** organic solar cells, non-fullerene acceptor, truxene, perylene diimides, selenium

## Abstract

An electron acceptor with a truxene core and ring-fusion perylene diimide (PDI) tripolymer annulated by selenium (Se) branch, named as FTr-3PDI-Se, is designed and synthesized. FTr-3PDI-Se exhibits large conjugated planar conformation, strong absorption spectra in the regions of 300–400 and 450–550 nm, the deep HOMO energy level of 6.10 eV, and high decomposition temperature above 400°C. The FTr-3PDI-Se: PBDB-T-2Cl based device achieved a disappointing power conversion efficiency (PCE) of 1.6% together with a high *V*_oc_ of 1.12 V. The low PCE was due to the large aggregates of blend film, the imbalanced hole/electron transport and low PL quenching efficiencies. The high *V*_oc_ can be attributed to the high-lying LUMO level of FTr-3PDI-Se and the low-lying HOMO level of PBDB-T-2Cl. Our research presents an interesting and effective molecule-designing method to develop non-fullerene acceptor.

## Introduction

Organic solar cells (OSCs) have attracted boundless interest over the past few decades owing to the advantages of light weight, low cost, wide source, and large-scale roll-to-roll printing process (Kang et al., 2016; Hou et al., 2018). Recently, the fullerene acceptors, due to their numerous of disadvantages of weak absorption, limited structural modifications and electronic energy levels non-tunability, were marginalized by non-fullerene acceptors (NFAs) (Cheng et al., 2018; Yan et al., 2018). Significant progress in NFAs-based OSCs has been achieved with power conversion efficiency (PCE) over 18% (Lin et al., 2020; Liu Q. et al., 2020; Zhan et al., 2021). Among the widely reported NFAs, fused-ring electron acceptors (FREAs) and perylene diimide derivatives (PDIs) are the two main study directions.

Because of the strong electron affinity, high absorption coefficient and electron mobility, as well as energy-level tunability, PDIs are widely developed (Zhan et al., 2011; Li and Wonneberger, 2012; Liu et al., 2016; Sun et al., 2016; Feng et al., 2018; Agnieszka and Frank, 2019; Li M. Y. et al., 2020). The large conjugated skeleton of PDI exhibits strong aggregation tendency, which may result in self-trapping of light excitons and afterwards generate fast bimolecular recombination of charge carriers, limiting the high performance of OSCs (Sharenko et al., 2013; Liu S. Y. et al., 2015). The researcher verified that changing the planarity of the PDIs is the popular methods to avoid this strong aggregation (Zhong et al., 2014, 2016; Lin et al., 2016; Zhang et al., 2016; Duan et al., 2017a; Liu X. et al., 2017; Liu et al., 2018). For example, various 3D electron acceptors with the central aromatic core (atom) and twisted PDI trimers or tetramer were investigated (Liu Y. H. et al., 2015; Lee et al., 2016; Zhan et al., 2017; Zhang A. D. et al., 2017; Lin et al., 2018a; Liu W. X. et al., 2020). A twisted configuration of PDIs is confirmed effectively to avoid large aggregation. However, the single bonding connection between central core and PDIs would weaken charge mobility due to an excessive twist geometry, giving a low OSCs performance. Therefore, the proper twisted non-planar structures, i.e., good balance of desirable film morphology with proper domain size and sufficient charge transport ability seems to be the key point for developing high-performance PDI electron acceptors (Lin et al., 2018a).

Interestingly, oxidative ring-fusion between the central aromatic core and the PDI branches was verified to be an effective strategy to achieve an exquisite balance aforesaid for high OSCs performance (Hartnett et al., 2016; Meng et al., 2016a, 2017; Zhong et al., 2016; Wang et al., 2017; Zhang J. Q. et al., 2017; Lin et al., 2018a; Chen et al., 2020). The fused PDI NFAs all exhibited better planarity than non-fused counterparts, since the aromatic core and PDI branches were locked by the adjacent benzene. Meanwhile, the fused PDI NFAs showed stronger intermolecular π-π stacking and higher electron mobility (Lin et al., 2018a). Moreover, these fused PDI NFAs generated proper phase separation with proper domain size and high domain purity when blended with donors (Chen et al., 2018; Hu et al., 2018; Wu et al., 2019). Therefore, the fused PDI NFAs displayed better OSCs properties compared with unfused ones (Li et al., 2016; Meng et al., 2016a, 2017; Liu X. F. et al., 2017; Wang et al., 2017; Zhang J. Q. et al., 2017; Lin et al., 2018a; Yin et al., 2019; Carlotti et al., 2020).

Recently, several studies showed that five-membered heteroatom-annulated (nitrogen/chalcogen-fused in bay regions) of PDIs has been regarded as the most effective molecular design strategy to achieve high performance OSCs (Sun et al., 2015; Meng et al., 2016a; Cann et al., 2017). The five-membered heteroatom-annulated PDI NFAs reinforced intra- and intermolecular interactions, leading to high electron mobility, which achieved improved PCEs. Among the varied nitrogen/chalcogen, the selenium atom (Se), since its enormous and loose electron cloud, is much easier to realize orbital overlap between the adjacent PDI NFAs, afterwards enhance the charge carrier mobility (Meng et al., 2016b; Li et al., 2018; Luo et al., 2018; Li G. et al., 2020; Yang et al., 2020). Moreover, due to the natural easy-polarizing characteristic of the Se atom, the Se-annulated PDIs exhibit the stronger intra- and intermolecular interactions, which also confirmed the important application foreground of Se-annulation PDIs in non-fullerene OSCs (Duan et al., 2017b; Yin et al., 2018; Li et al., 2019; Luo et al., 2019; Qureshi et al., 2020; Wang et al., 2020).

**Scheme 1:**
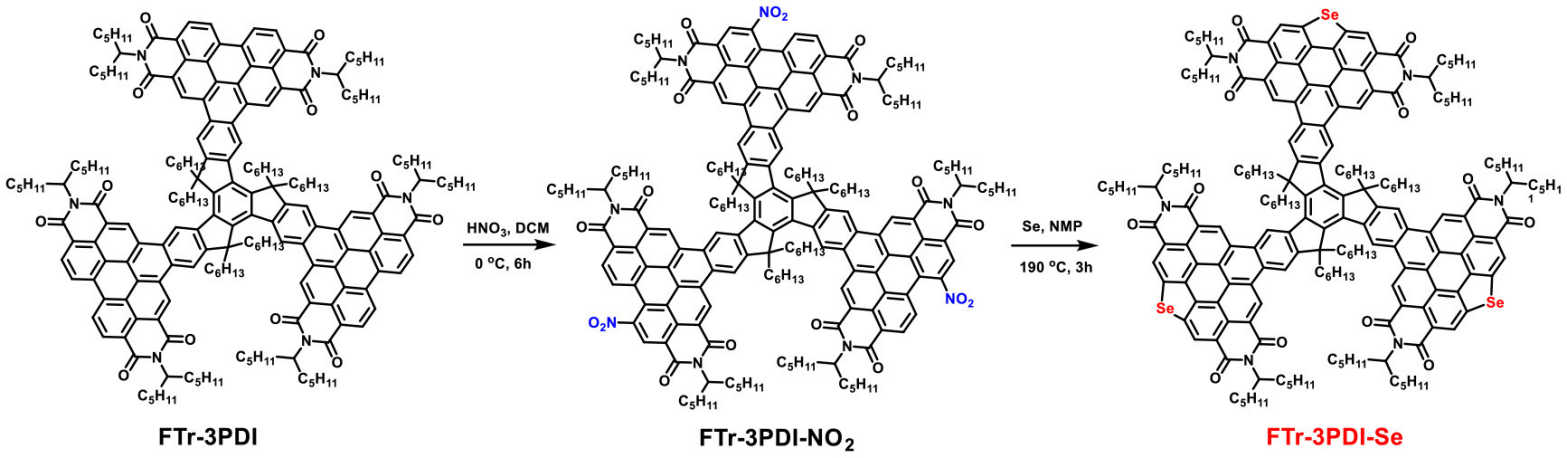
Chemical structure and synthetic routes of FTr-3PDI-Se.

Truxene has been demonstrated as a promising skeleton to construct high performance NFAs (Nielsen et al., 2013, 2014; Lin et al., 2018b; Wu et al., 2018). Inspired by the above achievements of Se-annulated PDIs, herein, we report the design and synthesis of truxene functionalized star-shaped NFAs with fused selenium-annulated PDIs, named FTr-3PDI-Se (Scheme 1). The devices based on poly[(2,6-(4,8-bis(5-(2-ethylhexyl-3-chloro)thiophen-2-yl)-benzo[1,2-b:4,5-b']dithiophene))-alt-(5,5-(1',3'-di-2-thienyl-5',7'-bis(2-ethylhexyl)benzo[1',2'-c:4',5'-c']dithiophene-4,8-dione)] (PBDB-T-2Cl): FTr-3PDI-Se exhibited a PCE of 1.6% with a high open-circuit voltage (*V*_oc_) of 1.12 V. The FTr-3PDI-Se exhibited large conjugated planar skeleton that can effectively promote the blend films to form large aggregates, which may lead to bimolecular recombination, limiting the OSCs performance.

## Result and Discussion

### Material Synthesis and Characterization

The synthetic routes of FTr-3PDI-Se was presented in Scheme 1 and the detailed synthetic procedure was provided in the Supporting Information. Compounds FTr-3PDI was synthesized according to the reported method (Lin et al., 2018a). FTr-3PDI-NO_2_ was prepared with a high yield of 95% using the fuming HNO_3_. Finally, the three fused selenium-annulated PDIs branches based on truxene, FTr-3PDI-Se, was synthesized by reductive cyclization reaction with Se powder. The as-synthesized FTr-3PDI-NO_2_ and FTr-3PDI-Se were fully characterized by ^1^H NMR, ^13^C NMR, and MALDI-TOF mass spectrometry (Supplementary Figures 1–6). Although large conjugated planar conformation, FTr-3PDI-Se electron acceptor displays moderate solubility in section of organic solvents such as chloroform, toluene, and chlorobenzene at room temperature. We ascribe it to the six hexyl chains of the truxene core.

### Theoretical Calculations

**Figure 1:**
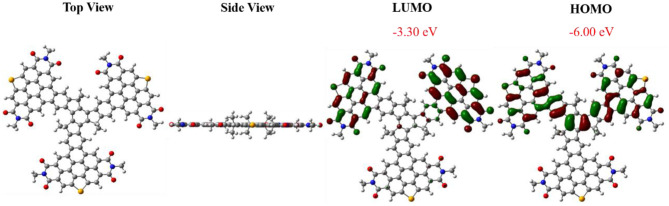
Views of the optimized geometries of FTr-3PDI-Se, and the LUMO/HOMO electron distribution obtained using DFT calculations at the B3LYP/6-31G(d) level.

The geometry and electron distribution of FTr-3PDI-Se was presented by employing the density functional theory (DFT) method at the B3LYP/6-31G(d,p) level in the Gaussian 09 software, where the long alkyl chain (–C_6_H_13_ of the truxene core and –C_5_H_11_ of the PDIs branches) was simplified to methyl groups (Figure 1). Obviously, FTr-3PDI-Se exhibits an overall planarity structure from the top view and side view. According to the optimized geometry, the highest occupied molecular orbital (HOMO) and lowest unoccupied molecular orbital (LUMO) electron distribution were calculated. The LUMO is distributed on two fused selenium-annulated PDIs sub-group. The HOMO is localized on one two fused selenium-annulated PDIs and truxene. The different wave function distributions between HOMO and LUMO are attributed to the degenerate orbital/multiple resonance configurations of the three fused selenium-annulated PDI groups. Furthermore, the calculated LUMO and HOMO levels were −3.30 and −6.00 eV.

### Thermodynamic, Optical, and Electrochemical Properties

**Figure 2:**
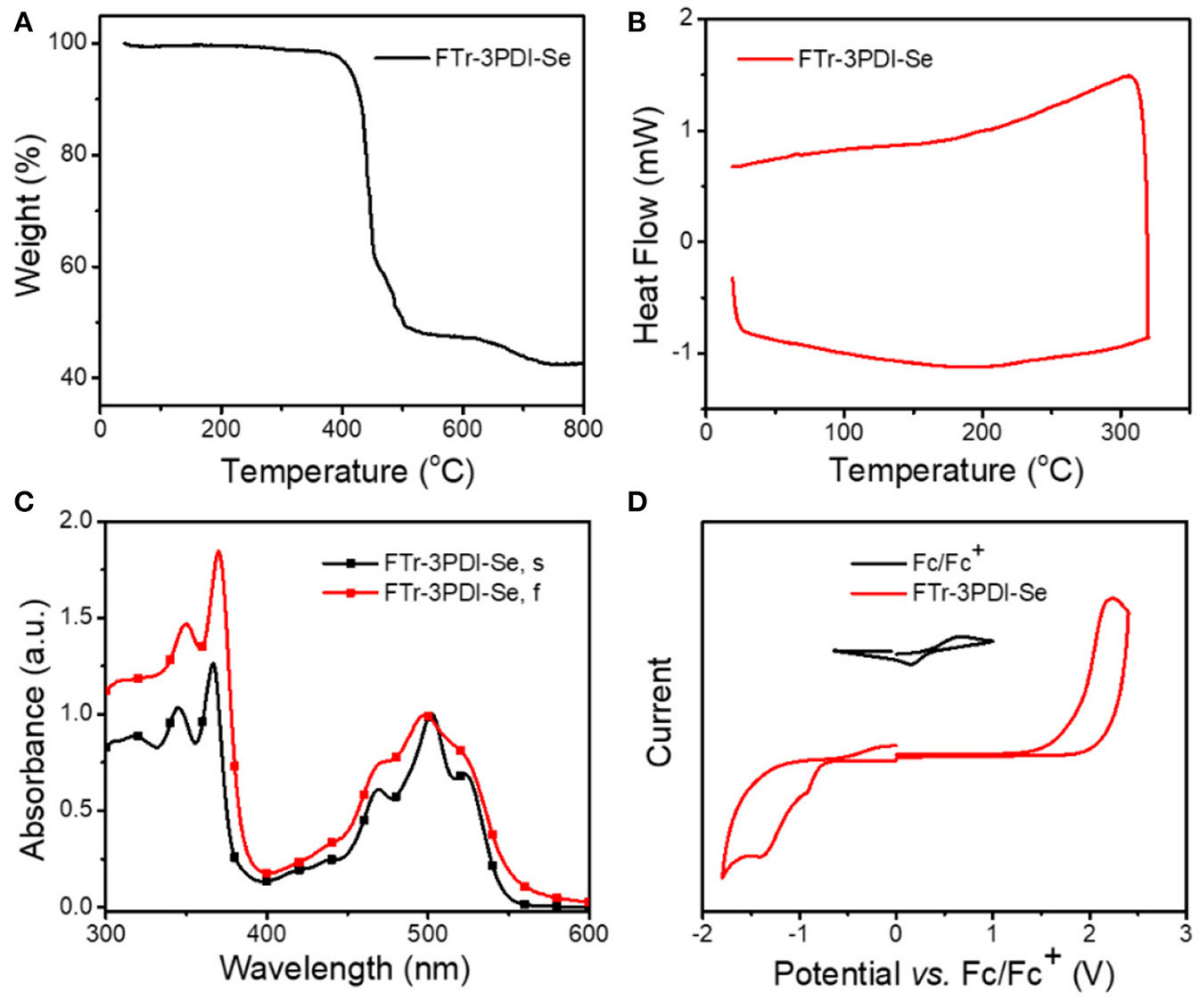
**(A)** TGA of FTr-3PDI-Se; **(B)** DSC of FTr-3PDI-Se; **(C)** normalized UV–vis absorption spectra of FTr-3PDI-Se in CHCl_3_ solution (FTr-3PDI-Se, s) and in film (FTr-3PDI-Se, f); **(D)** CV curves of FTr-3PDI-Se.

Thermogravimetric analysis (TGA) measurement (Figure 2A) demonstrated that FTr-3PDI-Se showed outstanding thermal stability along with a high decomposition temperature (*T*_d_, 5%weight loss) exceeding 400°C under nitrogen atmosphere, benefiting from large conjugated planar conformation. Afterwards, differential scanning calorimetry (DSC) was performed without obvious endo- and exothermal peaks from room temperature to 320°C in the second heating cycle (Figure 2B). The spectrum of FTr-3PDI-Se in chloroform solution showed two sets of absorption bands in the range of 300–600 nm. The short wavelength region displayed a maximal sharp peak of 360 nm with two broad shoulder peak, while the longer wavelength region exhibited the maximal peak of 500 nm with two broad shoulder peak as well (Figure 2C). FTr-3PDI-Se in thin film showed similar absorption spectra outline to their solution ones, indicating that the intermolecular aggregation is effective suppressed. Meanwhile, FTr-3PDI-Se demonstrated a slightly large optical bandgap of 2.24 eV with optical absorption onsets 555 nm ($E_{\text{g}}^{\text{opt}}$ = 1240/λ_onset_ eV). The absorption profiles of FTr-3PDI-Se is complementary to the strong absorption of PDBT-T-2Cl donor, which was exhibited in Supplementary Figure 7. The electrochemical property of FTr-3PDI-Se in chloroform solution was investigated by CV, as shown in Figure 2D. The half-wave potential of Fc/Fc^+^ was measured to be 0.40 V, and the energy levels of HOMO and LUMO were estimated from the onset oxidation ($E_{\text{ox}}^{\text{onset}}$) and reduction ($E_{\text{red}}^{\text{onset}}$) potentials by equations: *E*_HOMO_ = -*e*($E_{\text{ox}}^{\text{onset}}$ - *E*_Fc/Fc+_ + 4.8) and *E*_LUMO_ = -*e*($E_{\text{red}}^{\text{onset}}$ - *E*_Fc/Fc+_ + 4.8), respectively (Li et al., 1999). The HOMO/LUMO levels are −6.10/−3.65 eV. The slightly high-lying LUMO level cooperate with low-lying HOMO level of donor will contribute to achieve a high *V*_oc_. Meanwhile, the down-shifted HOMO level maintain the excellent chemical durability, and is favorable for hole transfer from excited acceptor to donor in OSCs (Duan et al., 2016, 2017b; 2018; Jia et al. 2017).

### Photovoltaic Properties

**Figure 3:**
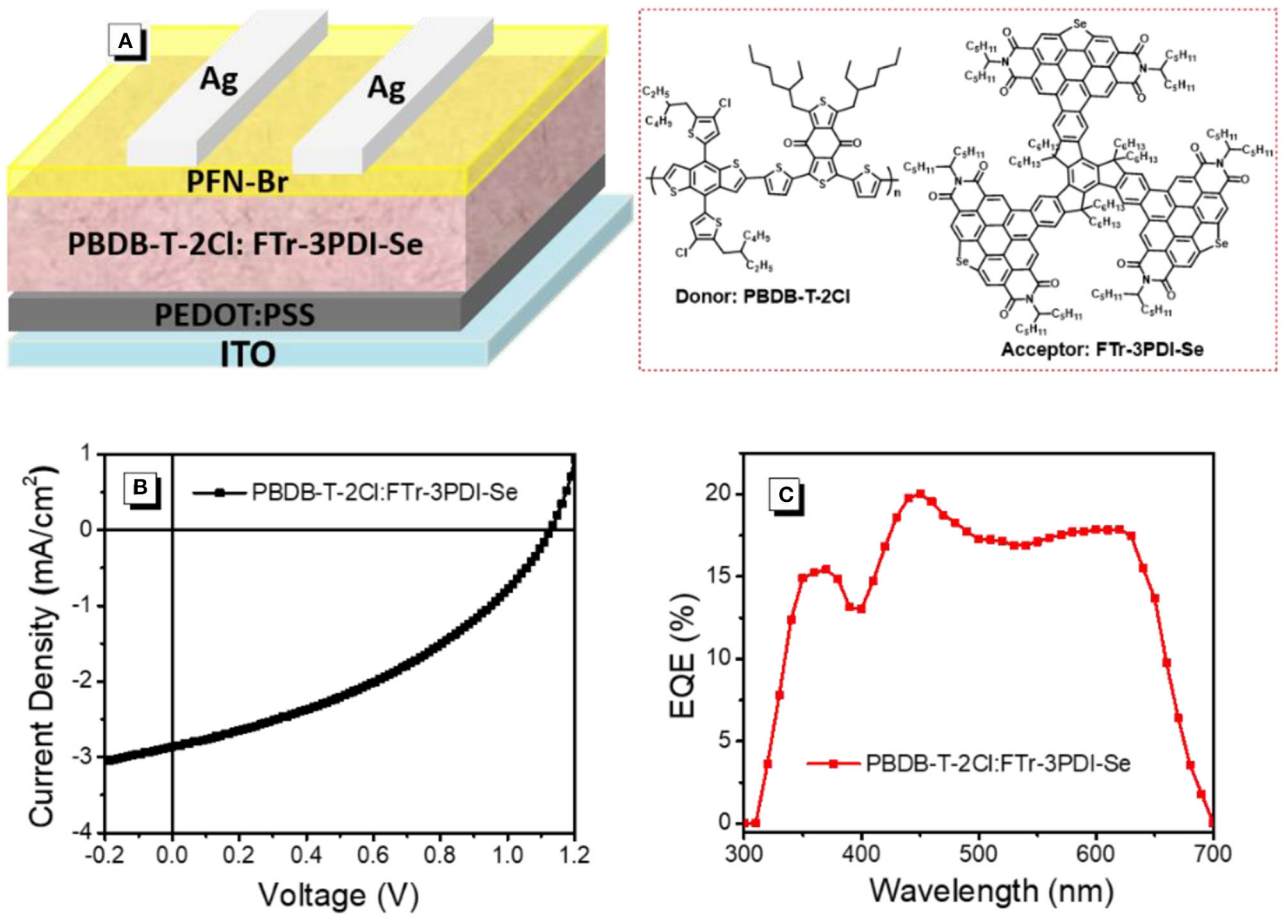
**(A)** The device structure; **(B)** current densityevoltage (*J*-*V*) characteristics; **(C)** external quantum efficiency (EQE) spectra of PBDB-T-2Cl: FTr-3PDI-Se solar cells.

The OSCs devices were prepared and measured with a conventional device structure of ITO (indiumtin oxide)/PEDOT:PSS (poly(3,4-ethylenedioxythiophene):poly (styrenesulfonate))/PBDB-T-2Cl: FTr-3PDI-Se/PFN-Br (poly[(9,9-bis(3'-((N,N-dimethyl)-N-ethylammonium)- propyl)-2,7-fluorene)-alt-2,7-(9,9-dioctylfluorene)])/Ag (Figure 3A). PDBT-T-2Cl was picked as the medium-bandgap donor to matched FTr-3PDI-Se acceptor benefiting from their complementary absorption and appropriate energy levels. The devices were fabricated and evaluated in terms of donor/acceptor weight ratios, solvent additives, and thermal annealing. All the device parameters under the mentioned above conditions are listed in Supplementary Tables 1–3. The optimal devices fabrication is that chlorobenzene as the main processing solvent with 1% chloronaphthalene solvent additives, and the annealing temperature is 120°C. The total concentration of PDBT-T-2Cl and FTr-3PDI-Se was optimized to be 20 mg mL^−1^ with the donor:acceptor weight ratio of 1.5:1. The optimized device parameters are summarized in Table 1, and the corresponding *J*–*V* curves are shown in Figure 3B. The optimized OSC device based on PBDB-T-2Cl: FTr-3PDI-Se exhibited a PCE of 1.6% with a high *V*_oc_ of 1.12 V, but a relatively poor short-circuit current density (*J*_sc_) of 3.6 mA cm^−2^ and a fill factor (FF) of 38.9%. The high *V*_oc_ is consistent with the high-lying LUMO level of FTr-3PDI-Se and low-lying HOMO level of PDBT-T-2Cl.

**Table 1:** Photovoltaic parameters of OSCs based on PBDB-T-2Cl: FTr-3PDI-Se under AM1.5G illumination at 100 mW cm^−2^.

**Acceptor devices**	***V*_**oc**_ (V)**	***J*_**sc**_ (mA cm^**−2**^)**	**$J_{\text{cal}}^{\text{a}}$ (mA cm^−2^)**	**FF**	**PCE (%)**
PBDB-T-2Cl: FTr-3PDI-Se	1.12	3.6	3.5	0.39	1.6

*^a^Calculated from EQE intetrations*.

The external quantum efficiency (EQE) spectra of PBDB-T-2Cl: FTr-3PDI-Se films were collected from the above optimized devices and displayed in Figure 3C. The calculated *J*_sc_ of 3.5 mA cm^−2^ from the EQE spectra was consistent with the measured *J*_sc_ (Table 1). The continuous EQE responses between 300 and 700 nm for the PBDB-T-2Cl: FTr-3PDI-Se based device results from the complementary absorption of PBDB-T-2Cl: FTr-3PDI-Se blend film (Supplementary Figure).

### Charge Transport and Recombination

The charge transport were acquired by single-carrier devices with a device structure of ITO/ZnO/PBDB-T-2Cl: FTr-3PDI-Se/Ca/Al for electron only devices and ITO/PEDOT:PSS/PBDB-T-2Cl: FTr-3PDI-Se/MoO_3_/Ag for hole only devices, respectively (Supplementary Figure 8). The hole mobilities (μ_h_) of PBDB-T-2Cl: FTr-3PDI-Se blend film was estimated to be 4.5 × 10^−6^ cm^2^ V^−1^ s^−1^. In contrast, the electron mobility (μ_e_) was measured to be 2.2 × 10^−4^ cm^2^ V^−1^ s^−1^, which are two orders of magnitude higher than μ_h_. The low hole mobility and highly imbalanced μ_e_/μ_h_ seriously suppress the charge transport and give rise to more bimolecular recombination, which in turn acquire low FF and *J*_sc_.

The photoluminescence (PL) quenching experiments were proceeded to study the charge transfer efficiency. As shown in Supplementary Figure 9, the PL quenching efficiencies of PBDB-T-2Cl: FTr-3PDI-Se blend films are 83.3 and 47.7% as compared to the neat PBDB-T-2Cl and FTr-3PDI-Se films, respectively, suggesting a moderate exciton dissociation efficiency.

### Morphology

**Figure 4:**
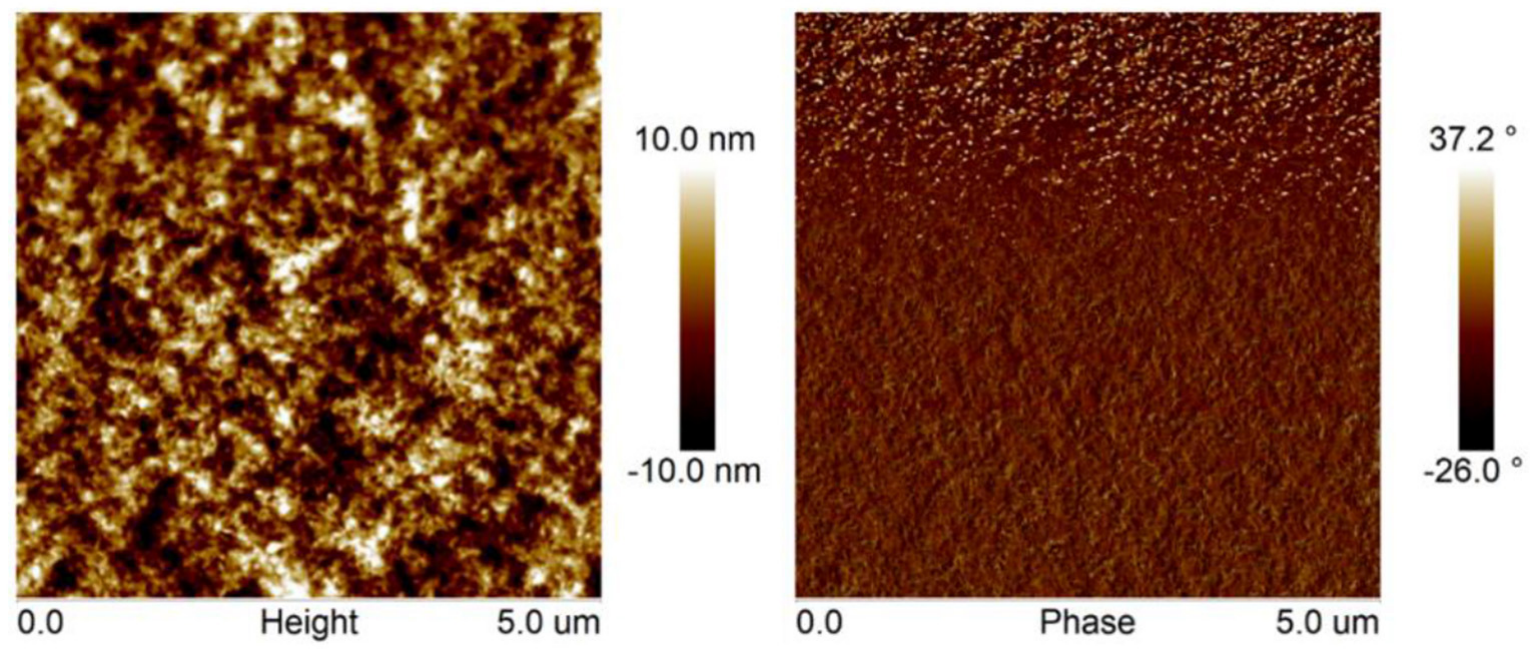
AFM height and phase images of PBDB-T-2Cl: FTr-3PDI-Se blend films.

The surface morphology of PBDB-T-2Cl: FTr-3PDI-Se blend films were investigated using atomic force microscopy (AFM). The film exhibited obvious phase separation with nanofibrillar structures (Figure 4), forming a relative coarse surface with a RMS surface roughness of 3.97 nm. The large planar conformation of FTr-3PDI-Se, can effectively promote the blend films to form large aggregates.

## Conclusion

In summary, FTr-3PDI-Se was synthesized and employed as electron acceptors for organic solar cells. The optimized devices based on PBDB-T-2Cl: FTr-3PDI-Se displayed a PCE of 1.6%, which was attributed to the following reasons. The conjugated planar conformation of FTr-3PDI-Se, verified by the DFT quantum calculation, can effectively promote the blend films to form large aggregates, which impeded the charge transport. Meanwhile, the imbalanced hole/electron transport and low PL quenching efficiencies seriously obstruct the charge transport and reduce exciton dissociation efficiency. Obviously, this research missed the balance between the highly twisted non-planar structures and coplanar conformation. Taking the excellent advantages into consideration and discard the disadvantages, we expect that the combination of the fused selenium-annulated PDIs with other conformation cores will create more promising and practical acceptors.

## Data Availability Statement

The original contributions presented in the study are included in the article/Supplementary Material, further inquiries can be directed to the corresponding authors.

## Author Contributions

KL and FH: designed experiments. KL, QY, ZW, BX, and YW: carried out experiments. KL, YC, and CD: analyzed experimental results. KL and CD: wrote the manuscript. All authors contributed to the article and approved the submitted version.

## Conflict of Interest

The authors declare that the research was conducted in the absence of any commercial or financial relationships that could be construed as a potential conflict of interest.

## References

[B1] Agnieszka N. K. Frank W. (2019). Progress in the synthesis of perylene bisimide dyes. Org. Chem. Front. 6, 1272–1318. 10.1039/C8QO01368C

[B2] Cann J. Dayneko S. Sun J.-P. Hendsbee A. D. Hill I. G. Welch G. C. (2017). N-Annulated perylene diimide dimers: acetylene linkers as a strategy for controlling structural conformation and the impact on physical, electronic, optical, and photovoltaic properties. J. Mater. Chem. C 5, 2074–2083. 10.1039/C6TC05107C

[B3] Carlotti B. Madu I. K. Kim H. Cai Z. X. Jiang H. J. Muthike A. K.. (2020). Activating intramolecular singlet exciton fission by altering p-bridge flexibility in perylene diimide trimers for organic solar cells. Chem. Sci. 11, 8757–8770. 10.1039/D0SC03271A PMC8163386 34123128

[B4] Chen F. Ding G. D. Tang A. L. Xiao B. Li J. F. Zhou E. J. (2018). A perylenediimide dimer containing an asymmetric p-bridge and its fused derivative for fullerene-free organic solar cells. J. Mater. Chem. C 6, 2580–2587. 10.1039/C8TC00089A

[B5] Chen H. Q. Wang L. Sun H. Liu Q. Tan X. Sang S. L.. (2020). PDI-based heteroacenes as acceptors for fullerene-free solar cells: importance of their twisted geometry. New J. Chem. 44, 13093–13099. 10.1039/D0NJ01733G

[B6] Cheng P. Li G. Zhan X. W. Yang Y. (2018). Next-generation organic photovoltaics based on non-fullerene acceptors. Nat. Photon. 12, 131–142. 10.1038/s41566-018-0104-929888443

[B7] Duan C. H. Guzám D. Colberts F. J. M. Janssen R. A. J. Torres T. (2018). Subnaphthalocyanines as electron acceptors in polymer solar cells: improving device performance by modifying peripheral and axial substituents. Chem. Eur. J. 24, 1–6. 10.1002/chem.201800596 29521455 PMC5947580

[B8] Duan C.H. Willems R. E. M. Franeker J. J. Bruijnaers B. J. Wienk. M. M Janssen. R. A. J. (2016). Effect of side chain length on the charge transport, morphology, and photovoltaic performance of conjugated polymers in bulk heterojunction solar cells. J. Mater. Chem. A, 4, 1855–1866. 10.1039/c5ta09483f

[B9] Duan Y. W. Xu X. P. Li Y. Li Z. J. Peng Q. (2017b). Chalcogen-atom-annulated perylene diimide trimers for highly efficient nonfullerene polymer solar cells. Macromol. Rapid Commun. 38:1700405. 10.1002/marc.201700405 28921741

[B10] Duan Y. W. Xu X. P. Yan H. Wu W. L. Li Z. J. Peng Q. (2017a). Pronounced effects of a triazine core on photovoltaic performance-efficient organic solar cells enabled by a PDI trimer-based small molecular acceptor. Adv. Mater. 29:1605115. 10.1002/adma.201605115 27922731

[B11] Feng J. J. Jiang W. Wang Z. H. (2018). Synthesis and application of rylene imide dyes as organic semiconducting materials. Chem. Asian J. 13, 20–30. 10.1002/asia.201701424 29143473

[B12] Hartnett P. E. Matte H. S. S. R. Eastham N. D. Jackson N. E. Wasielewski M. R. Marks T. J.. (2016). Ring-fusion as a perylenediimide dimer design concept for high-performance non-fullerene organic photovoltaic acceptors. Chem. Sci. 7, 3543–3555. 10.1039/C5SC04956C 29997846 PMC6007210

[B13] Hou J. H. Inganäs O. Friend R. H. Gao F. (2018). Organic solar cells based on non-fullerene acceptors. Nat. Mater. 17, 119–128. 10.1038/nmat5063 29358765

[B14] Hu H. W. Li Y. K. Zhang J. Q. Peng Z. X. Ma L. Xin J. M.. (2018). Effect of ring-fusion on miscibility and domain purity: key factors determining the performance of PDI-based nonfullerene organic solar cells. Adv. Energy Mater. 8:1800234. 10.1002/aenm.201800234

[B15] Jia J. C. Zheng N. N. Wang Z. F. Huang Y. P. Duan C. H. Huang F.. (2017). The effect of endcapping groups in A-D-A type non-fullerene acceptors on device performance of organic solar cells. Sci. China Chem. 60, 1458–1467. 10.1007/s11426-017-9102-1

[B16] Kang H. Kim G. Kim J. Kwon S. Kim H. Lee K. (2016). Bulk-heterojunction organic solar cells: five core technologies for their commercialization. Adv. Mater. 28, 7821–7861. 10.1002/adma.201601197 27345936

[B17] Lee J. Singh R. Sin D. H. Kim H. G. Song K. C. Cho K. (2016). A nonfullerene small molecule acceptor with 3D interlocking geometry enabling efficient organic solar cells. Adv. Mater. 28, 69–76. 10.1002/adma.201504010 26539752

[B18] Li C. Wonneberger H. (2012). Perylene imides for organic photovoltaics: yesterday, today, and tomorrow. Adv. Mater. 24, 613–636. 10.1002/adma.201104447 22228467

[B19] Li G. Wang S. H. Li D. D. Liu T. Yan H. Tang B.. (2020). Chalcogen-fused perylene diimides-based nonfullerene acceptors for high-performance organic solar cells: insight into the effect of O, S, and Se. Sol. RRL 4:1900453. 10.1002/solr.201900453

[B20] Li G. Wang S. H. Liu T. Hao P. Liu Z. H.. (2018). Non-fullerene acceptor engineering with threedimensional thiophene/selenophene-annulated perylene diimides for high performance polymer solar cells. J. Mater. Chem. C 6, 12601–12607. 10.1039/C8TC04926B

[B21] Li G. Yang S. F. Liu T. Li J. W. Yang W. B. Luo Z. H.. (2019). Functionalizing tetraphenylpyrazine with perylene diimides (PDIs) as high-performance nonfullerene acceptors. J. Mater. Chem. C 7, 14563–14570. 10.1039/C9TC05643B

[B22] Li M. Y. Yin H. Sun G. Y. (2020). PDI derivatives with functional active position as non-fullerene small molecule acceptors in organic solar cells: from different core linker to various conformation. Appl. Mater. Today 21:100799. 10.1016/j.apmt.2020.100799

[B23] Li S. X. Liu W. Q. Li C. Z. Liu F. Chen H. Z. Russelld T. P.. (2016). A simple perylene diimide derivative with a highly twisted geometry as an electron acceptor for efficient organic solar cells. J. Mater. Chem. A 4, 10659–10665. 10.1039/c6ta04232e

[B24] Li Y. F. Cao Y. Gao J. Wang D. L. Yu G. Heeger A. J. (1999). Electrochemical properties of luminescent polymers and polymer light-emitting electrochemical cells. Synth. Met. 99, 243–248. 10.1016/S0379-6779(99)00007-722887710

[B25] Lin H. R. Chen S. S. Hu H. W. Zhang L. Ma T. X. Yan H. (2016). Reduced intramolecular twisting improves the performance of 3D molecular acceptors in non-fullerene organic solar cells. Adv. Mater. 28, 8546–8551. 10.1002/adma.201600997 27501996

[B26] Lin K. W. Wang S. L. Wang Z. F. Yin Q. W. Liu X. Jia J. C.. (2018a). Electron acceptors with a truxene core and perylene diimide branches for organic solar cells: the effect of ring-fusion. Front. Chem. 6:328. 10.3389/fchem.2018.00328 30234096 PMC6131300

[B27] Lin K. W. Xie B. M. Wang Z. F. Duan C. H. Huang F. Cao Y.. (2018b). Star-shaped electron acceptors containing a truxene core for non-fullerene solar cells. Org. Electron. 52, 42–50. 10.1016/j.orgel.2017.10.009

[B28] Lin Y. Nugraha M. Firdaus Y. Scaccabarozzi A. Anies F. Emwas A.. (2020). A simple n-dopant derived from diquat boosts the efficiency of organic solar cells to 18.3%. ACS Energy Lett. 5, 3663–3671. 10.1021/acsenergylett.0c01949

[B29] Liu Q. Jiang Y. Jin K. Qin J. Xu J. Li W.. (2020). 18% Efficiency organic solar cells. Sci. Bull. 65, 272–275. 10.1016/j.scib.2020.01.001 36659090

[B30] Liu S. Y. Wu C. H. Li C. Z. Liu S. Q. Wei K. H. Chen H. Z.. (2015). A tetraperylene diimides based 3D nonfullerene acceptor for efficient organic photovoltaics. Adv. Sci. 2015, 2:1500014. 10.1002/advs.201500014 27980932 PMC5115352

[B31] Liu W. X. Zhang C. E. Liu J. C. Bo Z. S. (2020). PDI-based hexapod-shaped nonfullerene acceptors for the high-performance as-cast organic solar cells. ACS Appl. Mater. Interfaces 12, 37409–37417. 10.1021/acsami.0c11159 32814394

[B32] Liu X. Liu T. Duan C. H. Sun Y. M. Huang F. Cao Y.. (2017). Non-planar perylenediimide acceptors with different geometrical linker units for efficient nonfullerene organic solar cells. J. Mater. Chem. A 5, 1713–1723. 10.1039/C6TA08739F

[B33] Liu X. F. Cai Y. H. Huang X. B. Zhang R. B. Sun X. B. (2017). A perylene diimide electron acceptor with a triptycene core for organic solar cells. J. Mater. Chem. C 5, 3188–3194. 10.1039/C7TC00378A

[B34] Liu Y. H. Mu C. Jiang K. Zhao J. B. Li Y. K. Yan H.. (2015). A tetraphenylethylene core-based 3D structure small molecular acceptor enabling efficient non-fullerene organic solar cells. Adv. Mater. 27, 1015–1020. 10.1002/adma.201404152 25429918

[B35] Liu Z. T. Wu Y. Zhang Q. Gao X. (2016). Non-fullerene small molecule acceptors based on perylene diimides. J. Mater. Chem. A 4, 17604–17622. 10.1039/C6TA06978A

[B36] Liu Z. T. Zhang L. H. Shao M. Wu Y. Zeng D. Gao X.. (2018). Fine-tuning the quasi-3D geometry: enabling efficient nonfullerene organic solar cells based on perylene diimides. ACS Appl. Mater. Interfaces 10, 762–768. 10.1021/acsami.7b16406 29250948

[B37] Luo Z. H. Liu T. Chen Z. X. Xiao Y. Q. Yang C. L.. (2019). Isomerization of perylene diimide based acceptors enabling high-performance nonfullerene organic solar cells with excellent fill factor. Adv. Sci. 6:1802065. 10.1002/advs.201802065 30937273 PMC6425449

[B38] Luo Z. H. Liu T. Cheng W. L. Wu K. L. Xie D. J. Yang C. L.. (2018). A three-dimensional thiophene-annulated perylene bisimide as a fullerene-free acceptor for a high performance polymer solar cell with the highest PCE of 8.28% and a V_OC_ over 1.0 V. J. Mater. Chem. C 6, 1136–1142. 10.1039/C7TC05261H

[B39] Meng D. Fu H. T. Fan B. B. Li Y. Sun Y. M. Wang Z. H.. (2017). Rigid nonfullerene acceptors based on triptycene–perylene dye for organic solar cells. Chem. Asian J. 12, 1286–1290. 10.1002/asia.201700440 28422433

[B40] Meng D. Fu H. T. Xiao C. Y. Meng X. Y. Sun Y. M. Wang Z. H.. (2016a). Three-bladed rylene propellers with three-dimensional network assembly for organic electronics. J. Am. Chem. Soc. 138, 10184–10190. 10.1021/jacs.6b04368 27440216

[B41] Meng D. Sun D. Zhong C. M. Sun Y. M. Wang Z. H.. (2016b). High-performance solution-processed non-fullerene organic solar cells based on selenophene-containing perylene bisimide acceptor. J. Am. Chem. Soc. 138, 375–380. 10.1021/jacs.5b11149 26652276

[B42] Nielsen C. B. Voroshazi E. Holliday S. Cnops K. Cheyns D. McCulloch I. (2014). Electron-deficient truxenone derivatives and their use in organic photovoltaics. J. Mater. Chem. A 2, 12348–12354. 10.1039/C4TA01653J

[B43] Nielsen C. B. Voroshazi E. Holliday S. Cnops K. Randb B. P. McCulloch I. (2013). Efficient truxenone-based acceptors for organic photovoltaics. J. Mater. Chem. A 1, 73–76. 10.1039/C2TA00548D

[B44] Qureshi M. B. A. Li M. Wang H. Song J. S. Bo Z. S. (2020). Nonfullerene acceptors with an N-annulated perylene core and two perylene diimide units for efficient organic solar cells. Dyes Pigments 173:107970. 10.1016/j.dyepig.2019.107970

[B45] Sharenko A. Proctor C. M. Poll T. S. V. Henson Z. B. Nguyen T.-Q. Bazan G. C. (2013). A high-performing solution-processed small molecule: perylene diimide bulk heterojunction solar cell. Adv. Mater. 25, 4403–4406. 10.1002/adma.201301167 23788212

[B46] Sun D. Meng D. Cai Y. H. Huo L. J. Sun Y. M. Wang Z. H.. (2015). Non-fullerene-acceptor-based bulk-heterojunction organic solar cells with efficiency over 7%. J. Am. Chem. Soc. 137, 11156–11162. 10.1021/jacs.5b06414 26278192

[B47] Sun M. Müllen K. Yin M. Z. (2016). Water-soluble perylenediimides: design concepts and biological applications. Chem. Soc. Rev. 45, 1513–1528. 10.1039/C5CS00754B 26797049

[B48] Wang B. Liu W. Q. Li H. B. Mai J. Q. Li C. Z. Chen H. Z.. (2017). Electron acceptors with varied linkages between perylene diimide and benzotrithiophene for efficient fullerene-free solar cells. J. Mater. Chem. A 5, 9396–9401. 10.1039/C7TA02582C

[B49] Wang K. K. Xia P. Wang K. W. You X. X. Xia J. L.. (2020). π-Extension, selenium incorporation, and trimerization: “three in one” for efficient perylene diimide oligomer-based organic solar cells. ACS Appl. Mater. Interfaces 12, 9528–9536. 10.1021/acsami.9b21929 32009378

[B50] Wu M. L. Yi J.-P. Hu J. Xia P. Wang H. Chen F.. (2019). Ring fusion attenuates the device performance: star-shaped long helical perylene diimide based non-fullerene acceptors. J. Mater. Chem. C 7, 9564–9572. 10.1039/C9TC02150G

[B51] Wu W. L. Zhang G. J. Xu X. P. Wang S. C. Li Y. Peng Q. (2018). Wide bandgap molecular acceptors with a truxene core for efficient nonfullerene polymer solar cells: linkage position on molecular configuration and photovoltaic properties. Adv. Funct. Mater. 28:1707493. 10.1002/adfm.201707493

[B52] Yan C. Q. Barlow S. Wang Z. H. Yan H. Jen A. K.-Y. Marder. S. R.. (2018). Non-fullerene acceptors for organic solar cells. Nat. Rev. Mater. 3:18003. 10.1038/natrevmats.2018.3

[B53] Yang J. Chen F. Cong P. Xiao H. J. Geng Y. F.. (2020). Tuning the optoelectronic properties of vinylene linked perylenediimide dimer by ring annulation at the inside or outside bay positions for fullerene-free organic solar cells. J. Energy Chem. 40, 112–119. 10.1016/j.jechem.2019.03.007

[B54] Yin Y. L. Song J. Guo F. Y. Sun Y. M. Zhao L. C. Zhang Y. (2018). Asymmetrical vs. symmetrical selenophene-annulated fused perylenediimide acceptors for efficient non-fullerene polymer solar cells. ACS Appl. Energy Mater. 1, 6577–6585. 10.1021/acsaem.8b01484

[B55] Yin Y. L. Zheng Z. Chen D. Y. Liu M. Zhang J. Guo F. Y.. (2019). Fusion or non-fusion of quasi-two-dimensional fused perylene diimide acceptors: the importance of molecular geometry for fullerene-free organic solar cells. J. Mater. Chem. A 7, 27493–27502. 10.1039/C9TA10174H

[B56] Zhan L. L. Li S. X. Xia X. X. Li Y. K. Lu X. H. Zuo L. J.. (2021). Layer-by-layer processed ternary organic photovoltaics with efficiency over 18%. Adv. Mater. 33:2007231. 10.1002/adma.202007231 33598972

[B57] Zhan X. J. Xiong W. T. Gong Y. B. Liu T. Xie Y. J. Peng Q.. (2017). Pyrene-fused perylene diimides: new building blocks to construct non-fullerene acceptors with extremely high open-circuit voltages up to 1.26 V. Sol. RRL 1:1700123. 10.1002/solr.201700123

[B58] Zhan X. W. Facchetti A. Barlow S. Marks T. J. Ratner M. A. Wasielewski M. R.. (2011). Rylene and related diimides for organic electronics. Adv. Mater. 23, 268–284. 10.1002/adma.201001402 21154741

[B59] Zhang A. D. Li C. Yang F. Zhang J. Q. Wang Z. H. Wei Z. X.. (2017). An electron acceptor with porphyrin and perylene bisimides for efficient non-fullerene solar cells. Angew. Chem. Int. Ed. 56, 2694–2698. 10.1002/anie.201612090 28128532

[B60] Zhang J. Q. Li Y. K. Huang J. C. Hu H. W. Zhang G. Y. Yan H.e.. (2017). Ring-fusion of perylene diimide acceptor enabling efficient nonfullerene organic solar cells with a small voltage loss. J. Am. Chem. Soc. 139, 16092–16095. 10.1021/jacs.7b09998 29112393

[B61] Zhang X. Yao J. N. Zhan C. L. (2016). Synthesis and photovoltaic properties of low bandgap dimeric perylene diimide based non-fullerene acceptors. Sci. China Chem. 59, 209–217. 10.1007/s11426-015-5485-8

[B62] Zhong H. L. Wu C. H. Li C. Z. Carpenter J. Chueh C.-C. Jen A. K.-Y.. (2016). Rigidifying nonplanar perylene diimides by ring fusion toward geometry-tunable acceptors for high-performance fullerene-free solar cells. Adv. Mater. 28, 951–958. 10.1002/adma.201504120 26638861

[B63] Zhong Y.u. Trinh M. T. Xiao S. X. Ng F. Zhu X. Y. Nuckolls C.. (2014). Efficient organic solar cells with helical perylene diimide electron acceptors. J. Am. Chem. Soc. 136, 15215–15221. 10.1021/ja5092613 25313903

