# Supplementary material for: Truxene Functionalized Star-Shaped Non-fullerene Acceptor With Selenium-Annulated Perylene Diimides for Efficient Organic Solar Cells
Source: Front Chem. 2021 May 12;9:681994. doi: 10.3389/fchem.2021.681994 (PMC8149752; doi:10.3389/fchem.2021.681994)
Supplement: Supplementary file 1 [file Table_1.DOC]

Truxene Functionalized Star-shaped Non-fullerene Acceptor with Selenium-annulated Perylene Diimides for Efficient Organic Solar Cells

Kaiwen Lin^a,b^*****, Boming Xie^b^, Zhenfeng Wang^b^, Qingwu Yin^b^, Yuehui Wang^a^, Chunhui Duan^b^*****, Fei Huang^b^***** and Yong Cao^b^

^a^Department of Materials and Food, University of Electronic Science and Technology of China Zhongshan Institute, Zhongshan, 528402, P.R. China

^b^Institute of Polymer Optoelectronic Materials and Devices, State Key Laboratory of Luminescent Materials and Devices, South China University of Technology, Guangzhou 510640, P. R. China.

*Corresponding author, E-mail: [kevinlin1990@163.com](mailto:kevinlin1990@163.com); duanchunhui@scut.edu.cn; msfhuang@scut.edu.cn

Phone: (+)86-0760-88325742

**Table of Contents**

1. Instruments and characterization 2

2. Synthetic Procedures 3

3. NMR Spectra and MALDI-TOF-MS 5

4. Devices Fabrication 8

5. SCLC Measurements 10

6. PL quenching efficiency 11

7. Supplementary Information References 11

1. Instruments and characterization

^1^H and ^13^C NMR were characterized with Bruker-500 spectrometer in deuterated chloroform solution at 298 K. Chemical shifts were recorded as *δ* values (ppm) with the internal standard of tetramethylsilane (TMS). MALDI-TOF-MS was performed by using a Bruker Agilent1290/maXis impact. The geometry was optimized by density functional theory (DFT) calculations performed at the B3LYP/6-31G(d,p) level using the Gaussian 09. Thermogravimetric analyses (TGA) were performed on a Netzsch TG 209 under nitrogen at a heating rate of 10 ^o^C min^-1^. Differential scanning calorimetry (DSC) was performed on a Netzsch DSC 204 under nitrogen flow at heating/cooling rates of 10/20 ^o^C min^-1^. UV-vis absorption spectra were recorded on a HP 8453 pectrophotometer. Cyclic voltammetry (CV) was performed on a CHI600D electrochemical workstation with an ITO-coated glass working electrode and a Pt wire counter electrode at a scanning rate of 50 mV s^-1^ against an a saturated calomel electrode reference electrode with a nitrogen saturated anhydrous solution of tetra-*n*-butylammonium hexafluorophosphate in acetonitrile (0.1 mol L^-1^). Scanning electron microscopy (SEM) images were obtained on a JEOL JSM-6700F scanning electron microscope.

2. Synthetic Procedures

The monomers of FTr-3PDI was synthesized according to the reported procedures1-2. The synthetic route for FTr-3PDI-Se is showed in Scheme 1. The detailed synthesis procedures are described as following:

***Synthesis of*** ***FTr-3PDI-NO_2_***

A solution of FTr-3PDI (2.0 mmol) in CHCl_3_ was stirred at 0 °C for 15 min, then a diluted solution of fuming nitric acid (5 mL) in CHCl_3_ (15 mL) was added dropwise over 30 min. After stirring at 0 ^o^C for 2 h, water and CH_3_OH were added, then the mixture was extracted with chloroform three times. The combined organic layer was washed with water twice, and dried over anhydrous Na_2_SO_4_. The crude product was purified by silicon chromatography (petroleum ether (PE):ethyl acetate (EA), v/v (10:1)) to get a red solid with a yield of 95%. ^1^H NMR (CDCl_3_, 500 MHz): δ [ppm]: 10.64 (m, 9H), 9.69 (m, 3H), 9.20 (m, 3H), 9.06 (m, 3H), 8.93 (m, 3H), 5.42 (m, 6H), 3.80 (m, 6H), 3.16 (m, 6H), 2.45 (m, 12H), 2.08 (m, 12H) 1.46 (m, 70H), 1.38 (m, 12H), 0.93 (m, 74H), 0.36 (m, 18H). ^13^C NMR (125 MHz, CDCl_3_): δ [ppm]: 164.79, 162.73, 156.01, 149.67, 149.57, 148.08, 148.00, 142.06, 138.94, 130.09, 129.65, 129.48, 129.11, 128.17, 127.63, 127.37, 126.66, 125.57, 124.91, 124.42, 119.92, 117.90, 57.74, 55.78, 38.27, 32.00, 31.97, 31.52, 29.52, 22.82, 22.80, 22.30, 14.28, 14.24, 13.84, 13.82. MS (MALDI-TOF): calcd. for (C_201_H_237_N_9_O_18_), 3066.80; found, 3066.468.

***Synthesis of FTr-3PDI-Se***

In a 100 mL two-necked flask were placed 0.5 g selenium powder (15.6 mmol) and 40 mL of N-methylpyrrolidone under argon, then at 70 ^o^C 1-nitroperylene bisimide (1.56 mmol) was added. After stirring for 15 minutes, the mixture was heated at 190 ^o^C for 5 h. After cooled to room temperature, the mixture was poured into 500 mL of HCl (2M), then filtered and washed with water. The crude product was purified by silicon chromatography (PE:EA, v/v (10:1)) to get the desired compound as a red solid with a yield of 85%. ^1^H NMR (CDCl_3_, 500 MHz): δ [ppm]: 10.86 (m, 9H), 9.89 (m, 9H), 5.74 (m, 6H), 3.96 (m, 6H), 3.27 (m, 6H), 3.16 (m, 6H), 2.63 (m, 12H), 2.16 (m, 12H) 1.57 (m, 82H), 1.38 (m, 26H), 0.94 (m, 62H), 0.36 (m, 18H). ^13^C NMR (125 MHz, CDCl_3_): δ [ppm]: 166.11, 165.45, 164.96, 164.08, 155.59, 149.28, 141.78, 141.18, 140.96, 139.10, 134.42, 134.22, 130.23, 129.89, 129.28, 124.48, 124.12, 124.02, 123.91, 123.18, 122.96, 122.57, 120.43, 118.27, 58.64, 55.54, 53.57, 38.28, 32.08, 31.61, 29.85, 29.68, 27.06, 25.01, 22.88, 22.36, 18.60, 14.31, 14.27, 13.83. MS (MALDI-TOF): calcd. for (C_201_H_234_N_6_O_12_Se_3_), 3163.55; found, 3163.343.

3. NMR Spectra and MALDI-TOF-MS


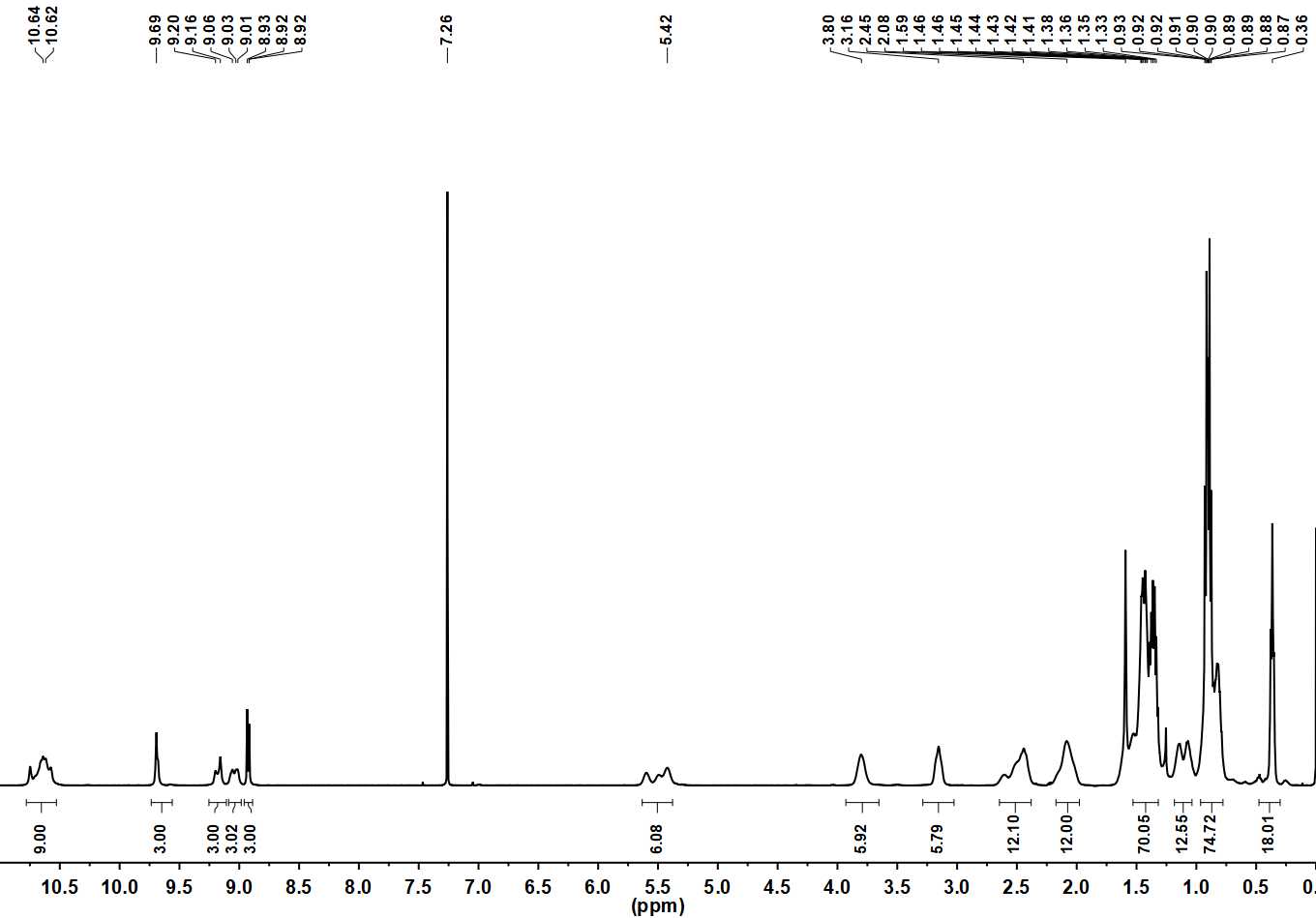


**Figure S1** ^1^H NMR spectrum of FTr-3PDI-NO_2_.


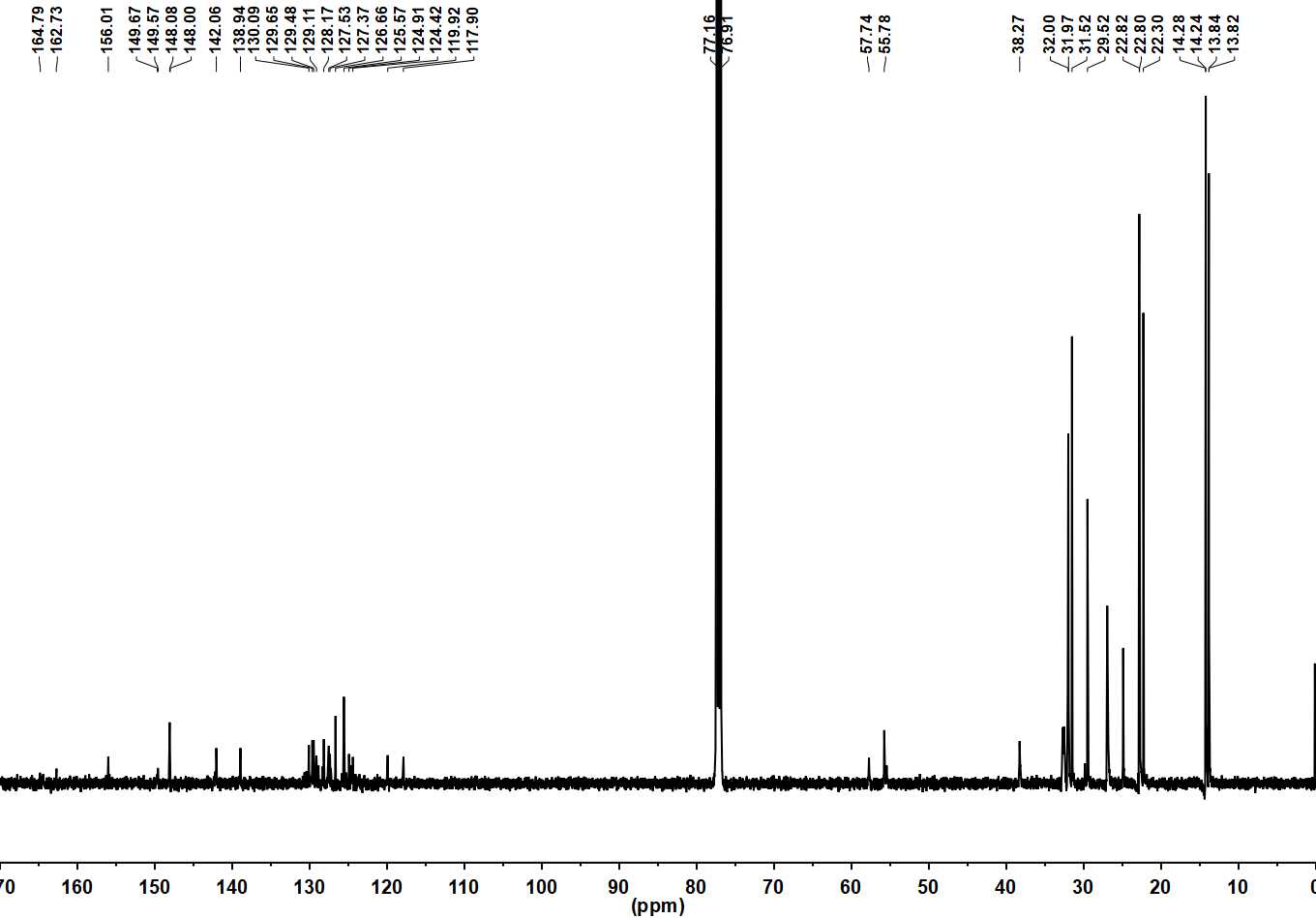


**Figure S2** ^13^C NMR spectrum of FTr-3PDI-NO_2_.


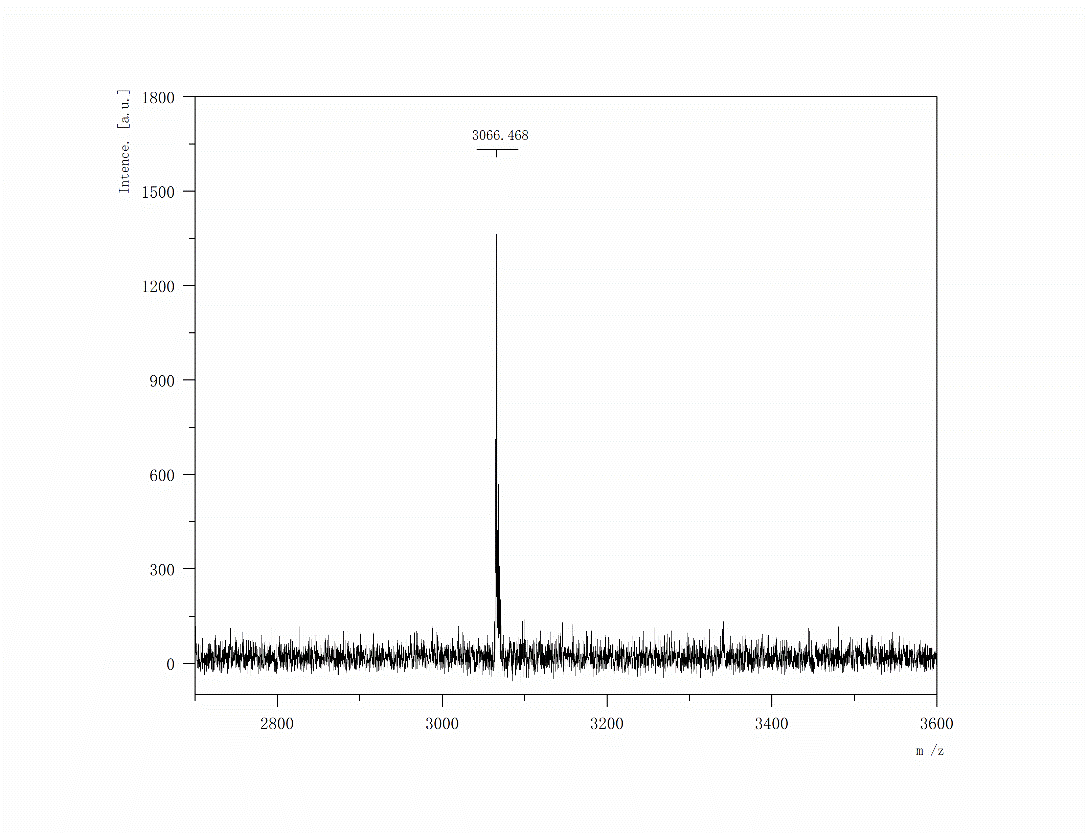


**Figure S3** MALDI-TOF-MS of FTr-3PDI-NO_2_.


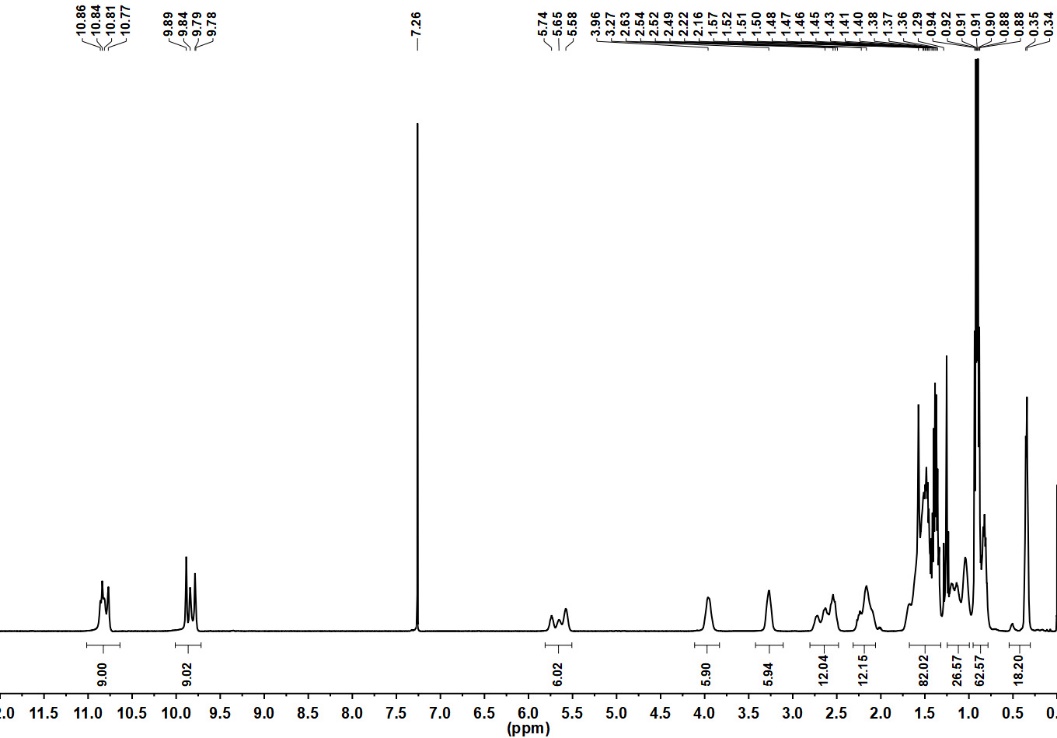


**Figure S4** ^1^H NMR spectrum of FTr-3PDI-Se.


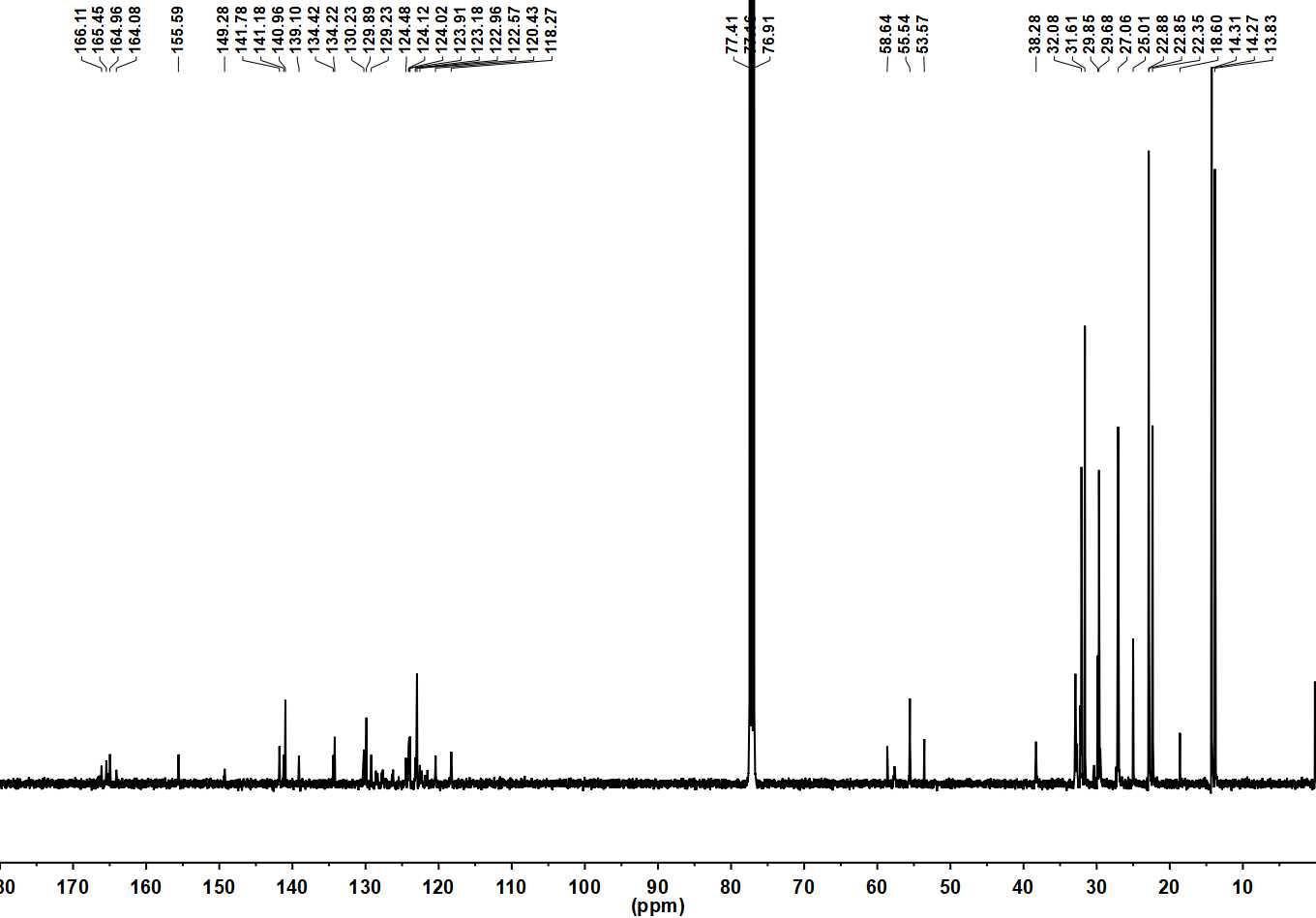


**Figure S5** ^13^C NMR spectrum of FTr-3PDI-Se.


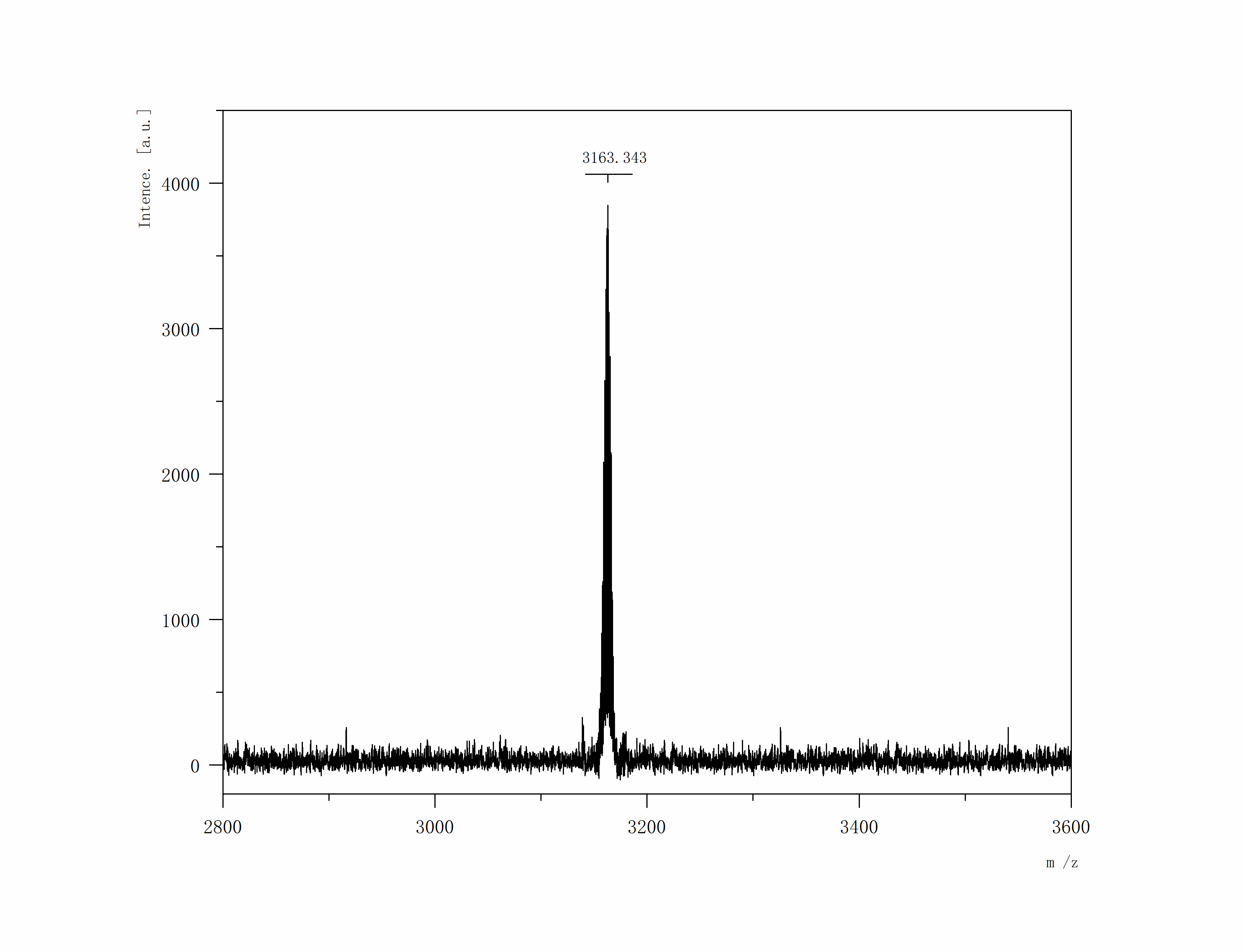


**Figure S6** MALDI-TOF-MS of FTr-3PDI-Se.

Figure S7 normalized UV–vis absorption spectra of PBDB-T-2Cl:FTr-3PDI-Se.

4. Devices Fabrication

The devices of indium tin oxide (ITO)/poly(3,4-ethylenedioxythiophene): poly(styrenesulfonate)(PEDOT:PSS)/ PBDB-T-2Cl: FTr-3PDI-Se /poly[(9,9-bis(3’-((N,N-dimethyl)-N-ethylammonium)-propyl)-2,7-fluorene)-*alt*-2,7-(9,9-dioctylfluorene)]dibromide (PFN-Br)/Ag were fabricated through the following procedures. The ITO-coated glass substrate was cleaned in an ultrasonic bath with deionized water, acetone, and isopropanol, each process was approximately 15 minutes, and then dried under a stream of dry nitrogen. PEDOT:PSS (Heraeus Clevios PVPA 4083) was spin-coated on top of the above ITO and annealed in air at 150 °C for 10 minutes. Subsequently, the blend solutions of PBDB-T-2Cl: FTr-3PDI-Se were prepared by simultaneously dissolving both materials with the optimized weight ratio in chlorobenzene and spin-coated on the ITO/PEDOT:PSS electrode (at 1600 rpm for 60 seconds) to form an active layer with thickness of about 100 nm. Then PFN-Br and Ag layer were thermally deposited onto the active layer through a shadow mask at a vacuum of 5×10^-5^ Pa. During the test, an aperture with an area of 3.14 mm^2^ was used. The current density–voltage (*J*–*V*) curves were measured on a computer-controlled Keithley 2400 source meter under 1 sun, the AM 1.5 G spectra came from a class solar simulator (Enlitech, Taiwan), and the light intensity was 100 mW cm^−2^ as calibrated by a China General Certification Center-certified reference monocrystal silicon cell (Enlitech). Before the *J*–*V* measurement, a physical mask with an aperture with precise area of 0.04 cm^2^ was used to define the device area. The external quantum efficiency (EQE) spectra were measured on a commercial QE measurement system (QE-R3011, Enlitech).

**TABLE S1** The optimization of donor/acceptor (D/A) weight ratios (the host solvents is CB; the thickness of active layers is 95 nm±5nm).

| Active layer | D/A ratio | Sol. Add. | Annealing | *V*oc  [V] | *J*sc [mA cm^–2^] | FF | PCE  [%] | Thickness  [nm] |
| --- | --- | --- | --- | --- | --- | --- | --- | --- |
| PBDB-T-2Cl: FTr-3PDI-Se | 1:1 | CB | 120℃10min | 1.07 | 1.04 | 31.48 | 0.35 | 95 |
|  | 1.5:1 | CB |  | 1.10 | 1.35 | 32.92 | 0.49 |  |
|  | 2:1 | CB |  | 1.05 | 1.14 | 31.51 | 0.39 |  |

**TABLE S2** The optimization of solvent additives (the host solvents is CB; D/A weight ratio is 1.5:1 for PBDBT-2Cl: FTr-3PDI-Se; the thickness of active layers is 95 nm±5nm).

| Active layer | D/A ratio | Sol. Add. | Annealing | Voc  [V] | Jsc [mA cm^–2^] | FF | PCE  [%] | Thickness  -nm] |
| --- | --- | --- | --- | --- | --- | --- | --- | --- |
| PBDB-T-2Cl: FTr-3PDI-Se | 1.5:1 | CB, 1%DIO | 120℃,10min | 1.12 | 1.09 | 32.48 | 0.40 | 95 |
|  |  | CB, 1%DPE |  | 1.08 | 1.00 | 30.21 | 0.33 |  |
|  |  | CB, 1%CN |  | 1.12 | 3.63 | 38.89 | 1.59 |  |
|  |  | CB, 1%ODT |  | 1.10 | 1.00 | 25.65 | 0.28 |  |

**TABLE S3** The optimization of thermal annealing (TA) for PBDBT-2Cl: FTr-3PDI-Se (the host solvents is CB; D/A weight ratio is 1.5:1; the thickness of active layers is 95 nm±5nm)

| Active layer | D/A ratio | Annealing | Sol. Add. | Voc  [V] | Jsc [mA cm^–2^] | FF | PCE  [%] | Thickness  [nm] |
| --- | --- | --- | --- | --- | --- | --- | --- | --- |
| PBDB-T-2Cl: FTr-3PDI-Se | 1.5:1 | 90℃ | CB, 1%CN | 1.12 | 3.52 | 37.45 | 1.48 | 95 |
|  |  | 120 |  | 1.12 | 3.63 | 38.89 | 1.59 |  |
|  |  | 150 |  | 1.11 | 2.36 | 35.58 | 0.93 |  |
|  |  | 180 |  | 1.10 | 2.44 | 37.37 | 0.99 |  |

CB, DIO, DPE, CN, and ODT are the abbreviation of chlorobenzene, 1,8-diiodooctane, diphenyl ether, chloronaphthalene, and octane-1,8-dithiol.

5. SCLC Measurements

**Electron Mobility Measurement by Space Charge Limited Current (SCLC) Method.**

Electron-only devices were fabricated by using the device structure of ITO/ZnO/ PDBT-T-2Cl:FTr-3PDI-Se /Ca/Ag. Device structures are ITO/ PEDOT:PSS/ PDBT-T-2Cl:FTr-3PDI-Se /MoO_3_/Ag for hole-only devices. And was measured by using the space-charge-limited current (SCLC) method. The mobility was calculated with the Mott−Gurney equation in the SCLC region: *J* = 9*ε*_0_*ε*_r_*μV*^2^/8*d*^3^, where *J* is the space charge limited current, *ε*_0_ is the permittivity of free space, *ε*_r_ is the relative permittivity of the material, *d* is the thickness of the material and *V* is the effective voltage. The effective voltage was obtained by subtracting the built-in voltage (*V*_bi_) and the voltage drop (*V*_s_) from the series resistance of the whole device except for the active layers from the applied voltage (*V*_appl_), *V* = *V*_appl_−*V*_bi_−*V*_s_. The electron mobility can be calculated from the slope of the *J*^1/2^~*V* curves.

**Figure S8** Space-charge-limited (SCLC) *J-V* characteristics of PDBT-T-2Cl:FTr-3PDI-Se under dark condition.

6. PL quenching efficiency


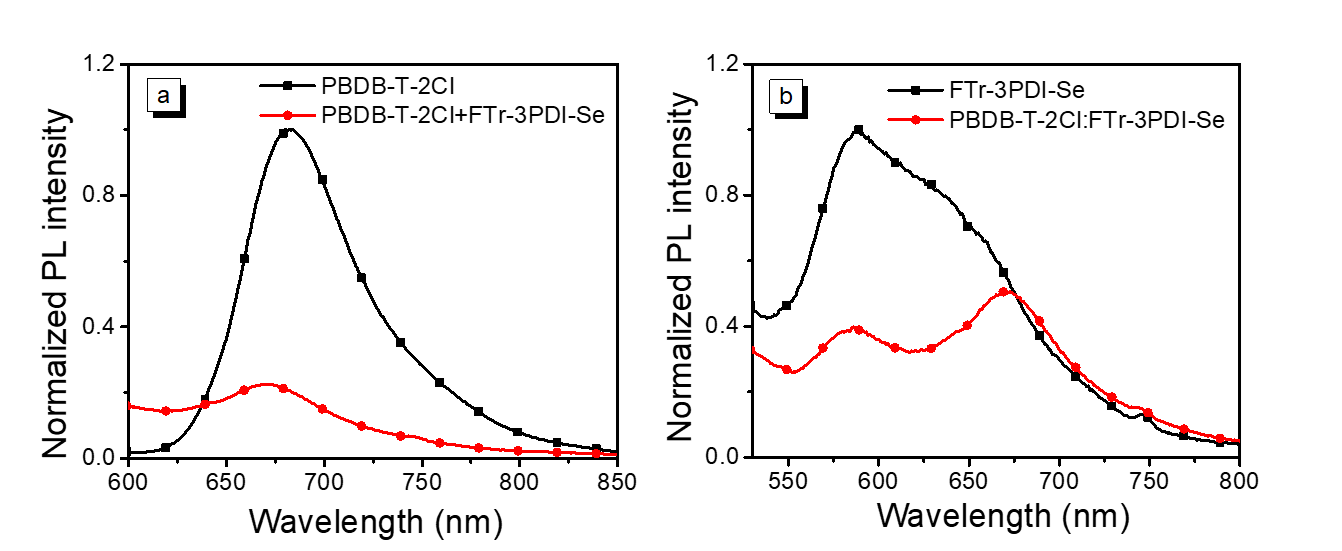


**Figure S9** PL quenching efficiency for donor/acceptor

7. Supplementary Information References

[1] Lin, K. W, Wang, S. L., Wang, Z. F., Yin, Q. W., Liu, X., Jia, J. C., Jia, X. E., Luo, P., Jiang, X. F., Duan, C. H., Huang, F., and Cao, Y. (2018) Electron acceptors with a truxene core and perylene diimide branches for organic solar cells: the effect of ring-fusion. Front. Chem. 6, 328. doi: 10.3389/fchem.2018.00328

[2] Lin, K. W., Xie, B. M., Wang, Z. F., Duan, C. H., Huang, F., Cao, Y., et al. (2018). Star-shaped electron acceptors containing a truxene core for non-fullerene solar cells. Org. Electron. 52, 42–50. doi: 10.1016/j.orgel.2017.10.009
